# Nematodal otitis by *Stenurus globicephalae* in a melon-headed whale (*Peponocephala electra*) stranded in Northeastern Brazil: case report and literature update

**DOI:** 10.1007/s11259-026-11370-6

**Published:** 2026-06-26

**Authors:** Hodias S. Oliveira Filho, José Lucas Costa Duarte, Raquel Mota Freitas Pereira, Pedro Lucas Jordão de Luna Araújo, Naftali Camily Fernandes de Lima, Raquel de Oliveira Simões, Bruno de Oliviera Telles Ferreira, Carlos Iberê Alves Freitas, Augusto Carlos da Bôaviagem Freire, Ana Bernadete Lima Fragoso, Flávio José de Lima Silva, Josué Díaz-Delgado, Jeann Leal de Araújo

**Affiliations:** 1 Laboratory of Conservation Medicine, Universidade Federal da Paraíba, Areia, Paraiba Brazil; 2 Projeto Cetáceos da Costa Branca, Universidade do Estado do Rio Grande do Norte, Areia Branca, Rio Grande do Norte Brazil; 3 Programa de Pós-graduação em Saúde Animal na Amazonia, Universidade Federal do Pará, Castanhal, Pará Brazil; 4 Laboratório de Patologia Animal, Universidade Federal do Pará, Castanhal, Pará Brazil; 5 Universidade do Estado do Rio Grande do Norte, Mossoró, Rio Grande do Norte Brazil; 6 Programa de Pós-graduação em Ciências Naturais, Universidade do Estado do Rio Grande do Norte, Mossoró, Brazil; 7 Programa de Pós-graduação Multicêntrico em Ciências Fisiológicas, Universidade do Estado do Rio Grande do Norte, Mossoró, Brazil; 8 Laboratório de Monitoramento de Biota Marinha, Universidade do Estado do Rio Grande do Norte, Mossoró, Rio Grande do Norte Brazil; 9 Laboratório de Diagnóstico Animal (LDA), Universidade Federal Rural de Pernambuco (UFRPE), Recife, Pernambuco Brazil; 10 Universidade Federal Rural do Rio de Janeiro (UFRRJ), Rio de Janeiro, Brazil; 11 Laboratório de Estudos em Imunologia e Animais Silvestres, Universidade Federal Rural do Semi-Árido (UFERSA), Mossoró, Rio Grande do Norte Brazil; 12 Texas Veterinary Medical Diagnostic Laboratory (TVMDL), College Station, TX USA; 13 Department of Veterinary Sciences, Federal University of Paraiba (UFPB), Areia, Paraiba 58397-000 Brazil

**Keywords:** Cetacean stranding, Marine mammal pathology, Tympanic bulla parasitism

## Abstract

Cetaceans are highly adapted marine mammals whose survival depends on precise auditory and echolocation systems. Therefore, disease processes affecting these systems, including parasitic infections pose significant morbidity and may result in stranding and death. Here, we describe the pathologic and parasitological findings in an adult, male melon-headed whale (*Peponocephala electra*) stranded dead on the Brazilian coast. The main gross pathologic findings consisted of bilateral otitis media associated with numerous nematodes in the tympanic bullae that occluded the tympanic lumen and resulted in compression of the tympanic membrane. Other relevant gross findings were abdominal effusion, visceral congestion, pulmonary petechiae, and lack of ingesta. Histopathologic examination of the tympanic membrane and middle ear soft tissue revealed chronic otitis and myringitis with epithelial hyperplasia and multifocal ulceration with intralesional nematodes. These nematodes were morphologically identified as *Stenurus globicephalae* through direct microscopy and electron microscopy analyses. In this case, severe parasitism of the tympanic apparatus was considered the primary pathologic condition. We surmise that the severity of the lesions noted in the auditory system could have resulted in disorientation and stranding, and, together with the physiological stress response associated with live stranding, ultimately led to death in this individual. This report underscores the role of parasitic otitis caused by *S. globicephalae* as a differential etiological diagnosis for stranding and death in cetaceans along the Brazilian coast and provides an updated overview of the literature on otic parasitism in cetaceans.

## Introduction

Cetaceans comprise 92 recognized species of whales and dolphins that occupy apex positions in marine trophic networks (Braulik et al. 2023). They are regarded as sentinel species, serving as robust bioindicators of marine ecosystem integrity and providing critical insights into pollutant burdens and ecological perturbations (Suárez-González et al. 2024; Esposito et al. 2025). The Brazilian coastline represents one of the longest Atlantic Ocean shorelines and supports at least 46 species of odontocetes reported in its waters (Miranda et al. 2020).

Marine echolocation is a particularly remarkable physiological specialization of cetaceans. Integrating complex sensory and cognitive structures, including the melon, mandible, brain, tympanic apparatus, and pterygoid sacs, cetaceans efficiently generate and emit sound pulses, process returning echoes, and localize objects within dynamic acoustic environments (Moss et al. 2023). This adaptation confers a substantial advantage in spatial orientation, prey detection, and precision hunting (Reidenberg and Laitman 2025). Evidence suggests that damage to one or more of these anatomic structures can have deleterious effects and may contribute to stranding events (Wang et al. 2021).

Melon-head whales (MHWs; *Peponocephala electra*) are relatively small and compact odontocetes, characterized by a rounded head, elongated pointed flippers, a curved falcate dorsal fin, and flukes with a concave trailing edge (Perryman and Danil 2018). They are often confused with the false killer whale (*Pseudorca crassidens*) and the pygmy killer whale (*Feresa attenuata*) (Phillips and Suepaul 2017). Living in tightly bonded social units, strandings of MHWs are of particular concern because single events may rapidly escalate into mass strandings, as demonstrated by one of the earliest reports of *P. electra* strandings in Brazil, dating back to 1897, which involved 240 individuals (Lodi et al. 1990). Although its conservation status is currently classified as Least Concern by the International Union for Conservation of Nature (IUCN 2025), melon-headed whales, as well as many other small cetacean species, face increasing anthropogenic pressures, including fisheries interactions, ocean noise, and habitat degradation, which may negatively affect population trends and conservation status in the future (Temple et al. 2024).

Parasitic infections are highly prevalent in free-ranging cetaceans, and multiparasitic infections are common across a wide range of species worldwide (Saldaña et al. 2022). Among these, trematodes (e.g., *Nasitrema* spp.) and nematodes (e.g., *Crassicauda* spp. and *Stenurus* spp.) frequently parasitize the tympano-periotic complex and pterygoid sacs of odontocetes and are often associated with high pathogenic potential (Suárez-González et al. 2024). *Stenurus* (Nematoda: Metastrongyloidea) are specialized parasites commonly reported in odontocetes worldwide. Eight species have been documented (Zylber et al. 2002), and their infection sites and host species can assist in presumptive diagnosis (Saldaña et al. 2022). The most common of these species are *S. minor*, which is largely encountered in harbor porpoises (*Phocoena phocoena*) (Wunschimann et al. 2001; Lehnert et al. 2005, 2017; Seibel et al. 2010; Morell et al. 2017; Wohlsein et al. 2019); S. *globicephalae*, typically reported in short-finned pilot whales (*Globicephala macrorhynchus*) (Carvalho et al. 2010; Saldaña et al. 2022), long-finned pilot whales (*G. melas*) (Abollo et al. 1998; Lehnert et al. 2017), Risso’s dolphins (*Grampus griseus*) (Lehnert et al. 2017) and *P.* *electra* (Carvalho et al. 2010), and *Stenurus ovatus*, which causes verminous pneumonia in striped dolphins (*Stenella coeruleoalba*) (Pool et al. 2020) and bottlenose dolphins (*Tursiops truncatus*) (Kuwamura et al. 2007; Tomo et al. 2010). Parasitic burns of *Stenurus* spp., particularly when affecting cranial sinuses or auditory structures, have the potential to interfere with echolocation, a critical function for cetacean navigation (Moss et al. 2023), leading to aberrant behavior and, ultimately, stranding events (Mann et al. 2010).

Here, we report a case of a melon-headed whale presenting with severe bilateral otitis media caused by *Stenurus globicephalae* that resulted in live stranding followed by death. We describe the gross, histopathological, and ultrastructural findings and integrate these observations with a critical review of the available literature.

## Case presentation

**Fig. 1:**
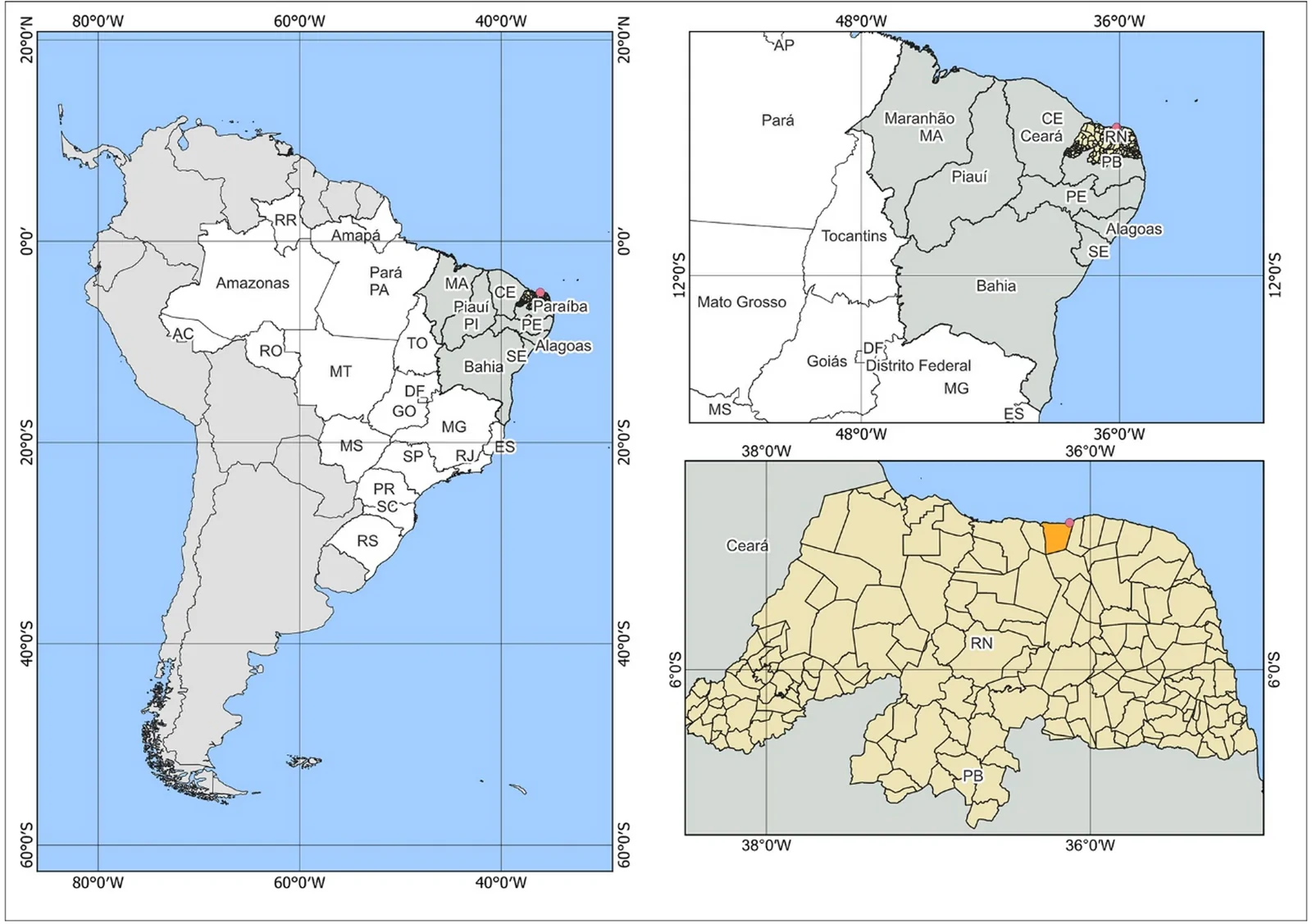
Geographic location of the stranding of a melon-headed whale (*Peponocephala electra*) on Catavento beach, Galinhos, Northeastern Brazil. (Lat: [-5.09231], Long: [-36.12776])

This case resulted from the efforts of the “Programa de Monitoramento de Praias da Bacia Potiguar” (PMP-BP; Beach Monitoring Program of the Potiguar Basin). During daily monitoring, a free-ranging, adult MHW was found stranded alive and died shortly after on Catavento Beach, Galinhos, Brazil (*Lat: -5.09231 Long: -36.12776*) (Fig. 1). Local residents reported that another individual had been sighted nearby, however, only one cetacean was ultimately found.

The cadaver was transported to the “Laboratório de Monitoramento de Biota Marinha, Projeto Cetáceos da Costa Branca” (PCCB-UERN). The necropsy was performed in accordance with internal guidelines for cetacean postmortem investigation and tissue sampling, eight hours after carcass recovery. At necropsy, samples of the heart, liver, kidneys, urinary bladder, lymph nodes, lungs, trachea, great vessels, peritoneal adipose tissue, skeletal muscle, and brain were fixed in 10% neutral buffered formalin and subsequently stored in 70% ethanol. The tissues were embedded in paraffin blocks, sectioned at 4 μm-thick, and routinely stained with hematoxylin and eosin (H&E) for histopathological evaluation. The flippers and skull were removed and submitted for radiographic evaluation. The age class was estimated based on total body length and skeletal ossification, observed through radiographic analysis of the flippers (Roca-Monge et al. 2022).

To access the tympanic and periotic bones (tympano-periotic complex, middle and inner ear, respectively), the lower jaw was disarticulated. Subsequently, the tympano-periotic complex was gently fractured for examination of the internal structures. Parasites observed inside were photographed with macro lens and sent for complete parasitological evaluation to the department of parasitology at the Universidade Federal Rural do Rio de Janeiro, preserved in AFA solution (alcohol, formalin, and acetic acid) (Carvalho et al. 2010). The exact number of nematodes present in the tympanic apparatus was not determined. The tympanic apparatus was initially fixed intact in 10% neutral buffered formalin to posterior histopathological evaluation.

For parasitological identification of nematodes from the tympanic apparatus, specimens were cleared in Amann’s lactophenol to allow for detailed visualization of internal structures under light microscopy Olympus BX51. Species confirmation was based on taxonomic keys and on morphological and morphometric comparison with key literature, including the faunistic catalogue for Brazil (Pinto et al. 2011) and the detailed redescriptions provided in other studies (Zylber et al. 2002; Shamsi et al. 2024). For scanning electron microscopy (SEM) preparation, specimens were dehydrated through a graded ethanol series, critical point dried with carbon dioxide, coated with gold, and examined using a JEOL™ JSM-6390 microscope (Tokyo, Japan) at the Rudolf Barth Electron Microscopy Platform of Oswaldo Cruz Institute (IOC), Rio de Janeiro, Brazil.

**Fig. 2:**
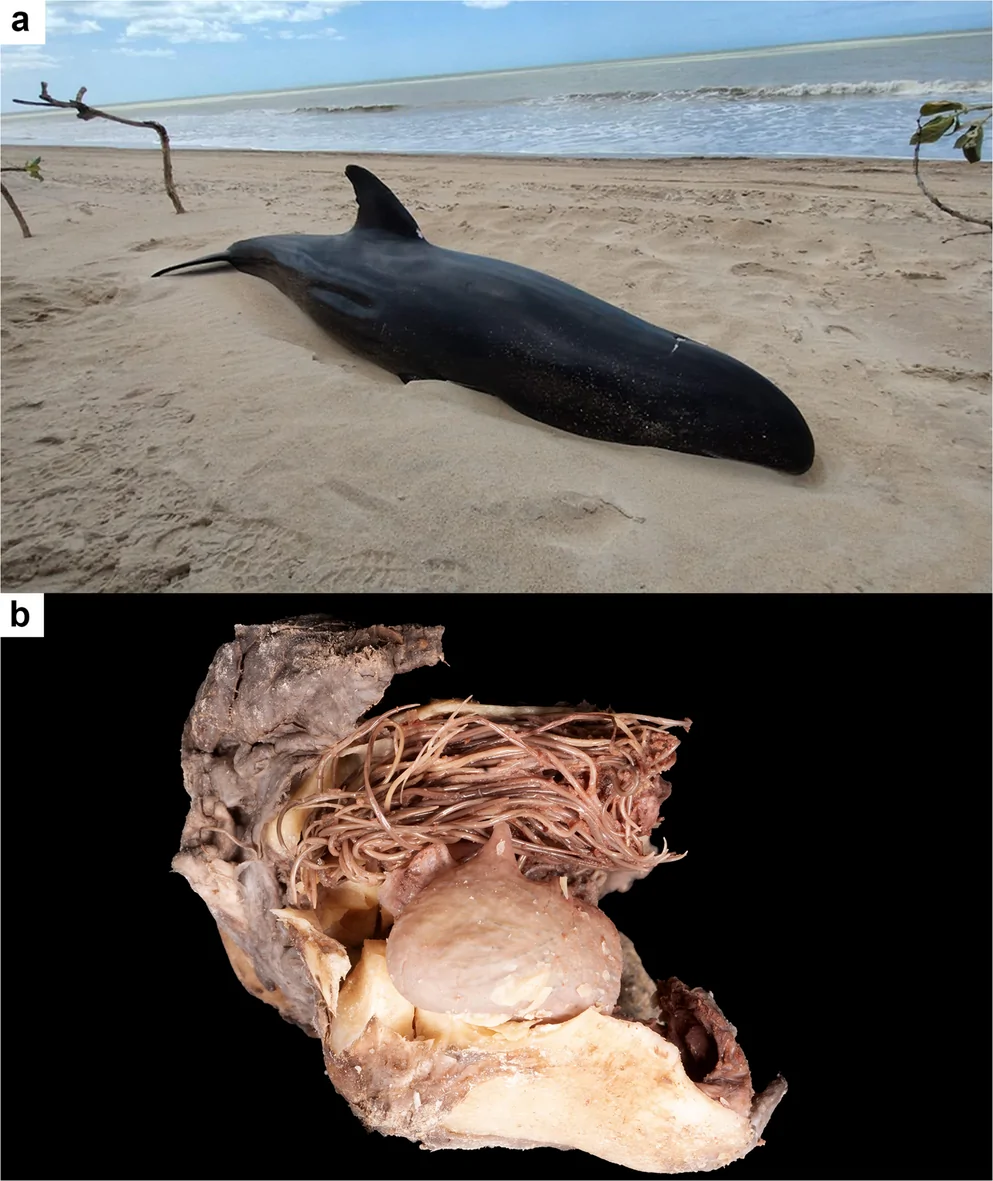
Field and necropsy findings of a melon-headed whale (*Peponocephala electra*) with severe nematodal otitis. **(a)** The cadaver presented a poor body condition. **(b)** Myriads of thin, filiform nematodes (*Stenurus globicephalae*) were densely packed, obstructing the lumen and compressing the tympanic membrane

**Fig. 3:**
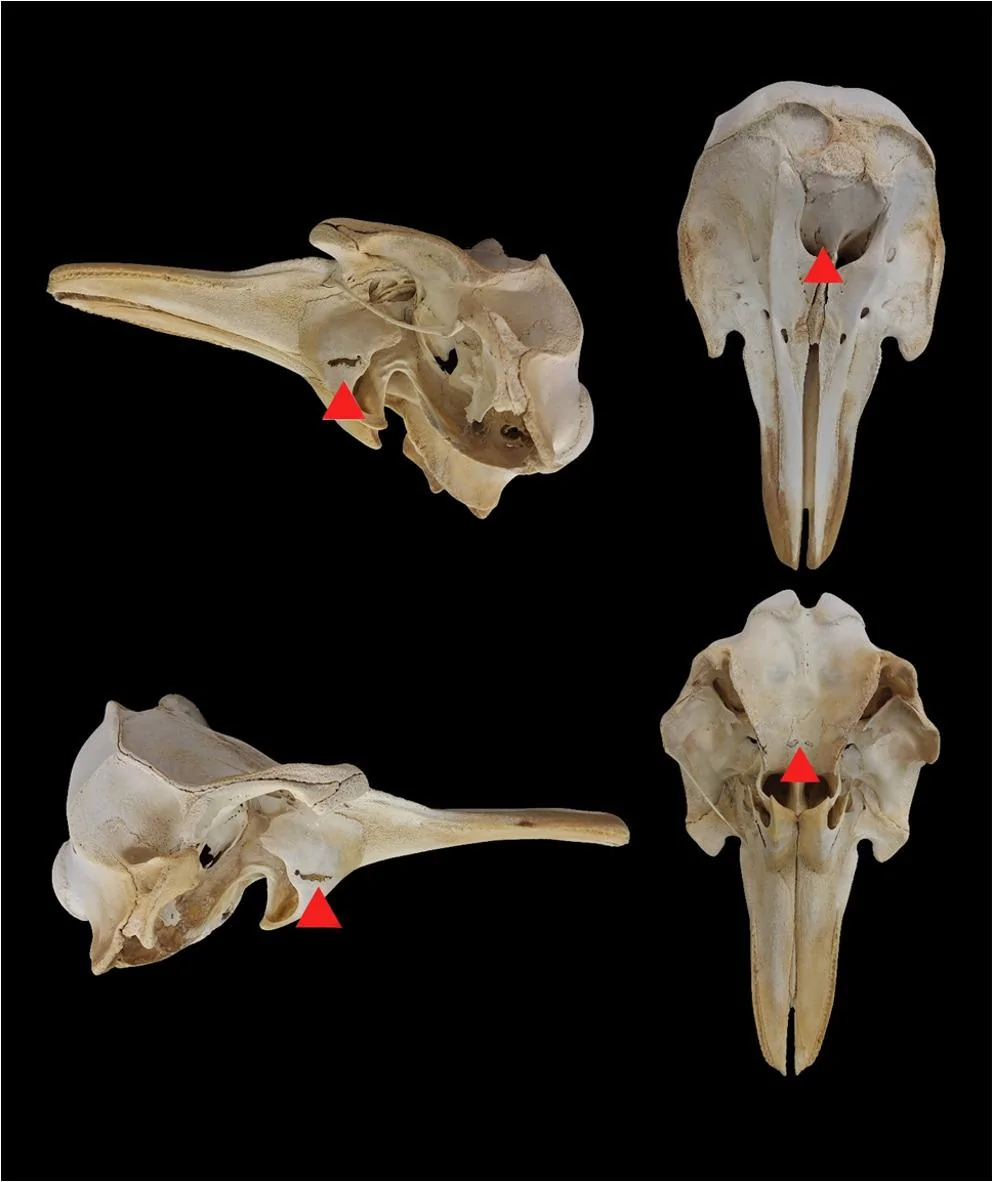
Osteolytic lesions in the skull of a melon-headed whale (*Peponocephala electra*) with nematodal otitis by *Stenurus globicephalae*. The lesions range from circular to irregular fenestrations of the basioccipital, and pterygoid bones (arrowheads)

The animal was a 2.5 m-long, ~ 150 kg, adult male, in poor body condition (Fig. 2A). Bilaterally, the conjunctiva were congested. Examination of the pterygoid sinuses and the tympano-periotic complexes revealed numerous filiform nematodes measuring approximately 37–45 mm in length, densely packed as a single mass in the tympanic bullae, causing compression of the tympanic membrane and complete obstruction of the lumens (Fig. 2B). Multifocal 1 to 4 cm in diameter, round to irregular fenestrations (osteomyelitis, osteolysis) were seen bilaterally in basioccipital, and pterygoid bones (Fig. 3). There was moderate serosanguineous effusion in the peritoneal cavity, visceral congestion, including prominent congestive splenomegaly, and petechiae along the visceral pleurae. The gastric compartments lacked ingesta.

**Fig. 4:**
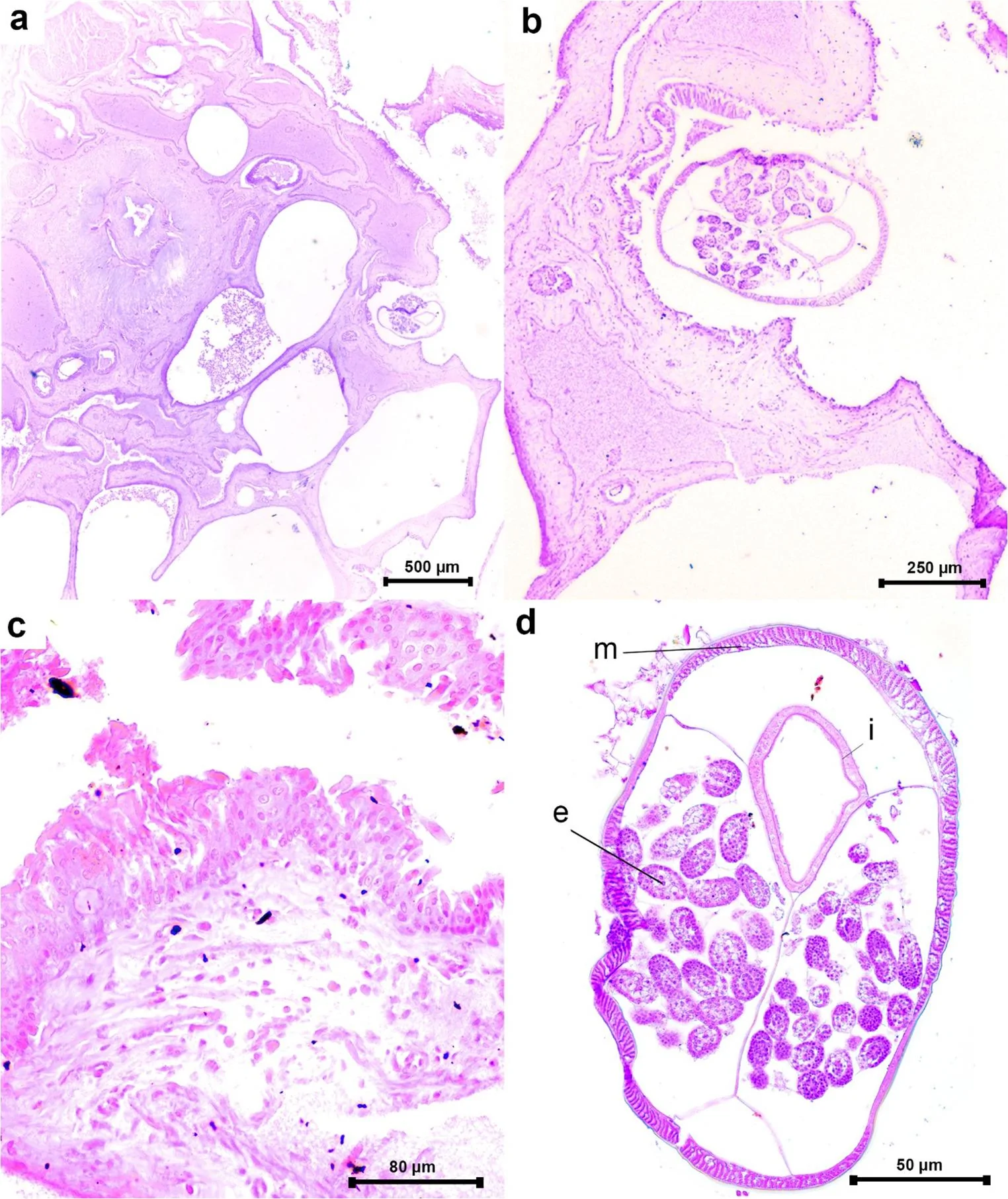
Microscopic findings of *Stenurus globicephalae* associated with severe otitis in a melon-headed whale (*Peponocephala electra*). **a-b.** Tympanic membrane showing multiple transverse sections of nematodes within the tympanic cavity lumen and mucosal cavitations. Hematoxylin and eosin (HE), objective 4X and 10X. **c.** Middle ear epithelium exhibits hyperplasia and intra- and intercellular edema. The subepithelial connective tissue has increased clear space (edema) and pleocellular inflammatory cells. HE, objective 40X. **d.** Transverse section of *S. globicephalae* demonstrating a thin cuticle, coelomyarian musculature (m), and a prominent uterus filled with embryonated eggs (e). A segment of intestine lined by uninucleated cuboidal cells (i) is noted. HE, objective 40X

Microscopically (Fig. 4), the tympanic membrane and middle-ear soft tissue exhibited marked epithelial hyperplasia with frequent intra- and intercellular edema and multifocal erosion and ulceration. The subepithelial connective tissue had scattered foci of viable and degenerated granulocytes, as well as edema and multifocal fibrosis. The vasculature was moderately to markedly dilated (erythema, congestion). The intraluminal adult nematodes had a thin cuticle, pseudocoelom, moderate coelomyarian musculature and digestive and reproductive structures. Uterine segments were frequently filled with embryonated eggs (Fig. 4D).

**Fig. 5:**
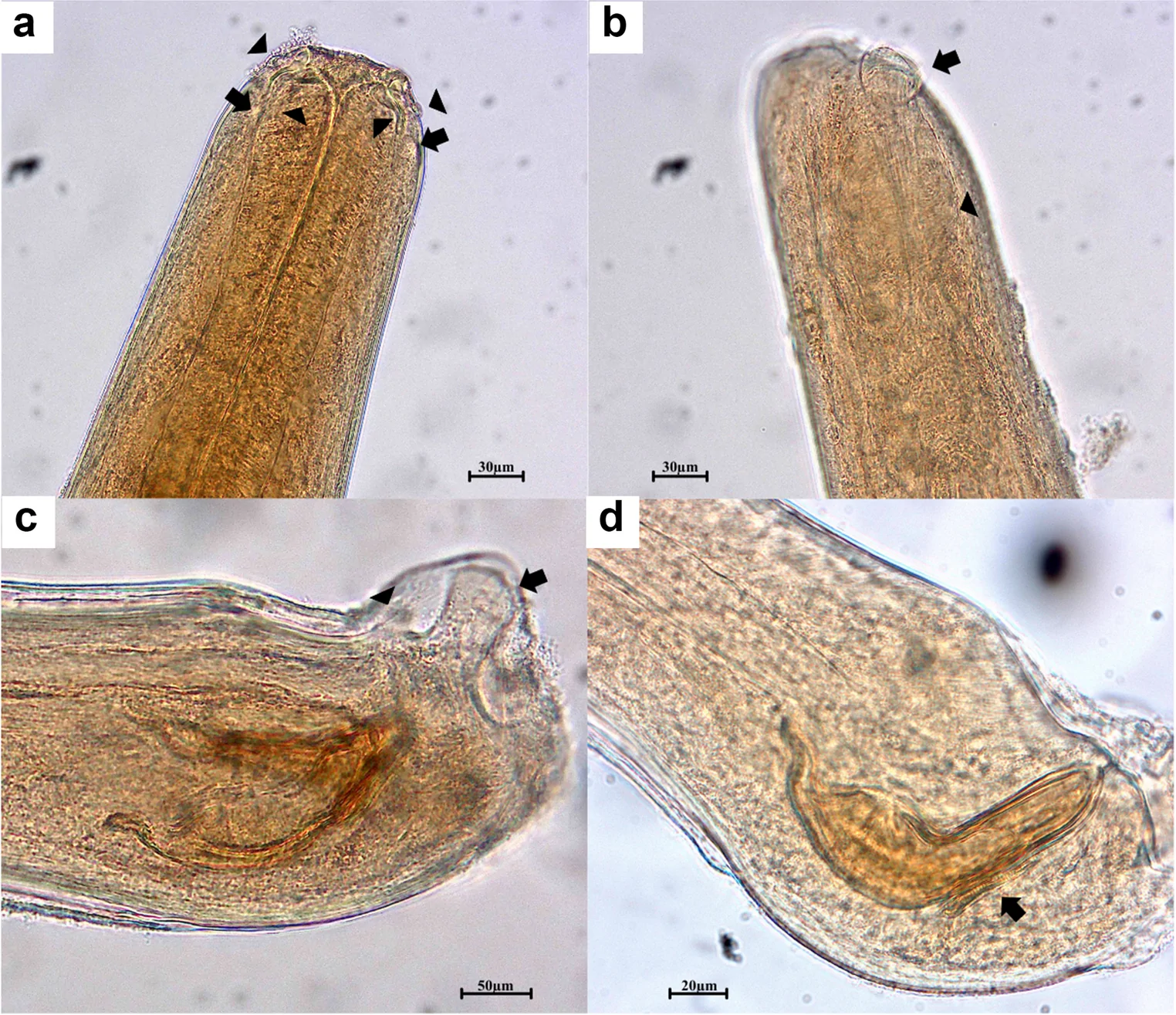
Light photomicrographs of *Stenurus globicephalae*. **(a)** Lateral view of the anterior end of a male showing the funnel-shaped buccal cavity (arrowheads) and the arrangement of cephalic papillae (arrows). **(b)** Lateral view of the caudal end of a female, showing the elongated vaginal sphincter (arrowhead) and the characteristic spherical cuticular swelling surrounding the vulva (arrow). **(c)** Ventrolateral view of the caudal end of a male, showing the copulatory bursa with its lateral ray (arrow), medial papilla (arrowhead) and the curved, equal spicules. **(d)** Higher magnification of the male caudal end, showing the simple gubernaculum (arrow)

**Fig. 6:**
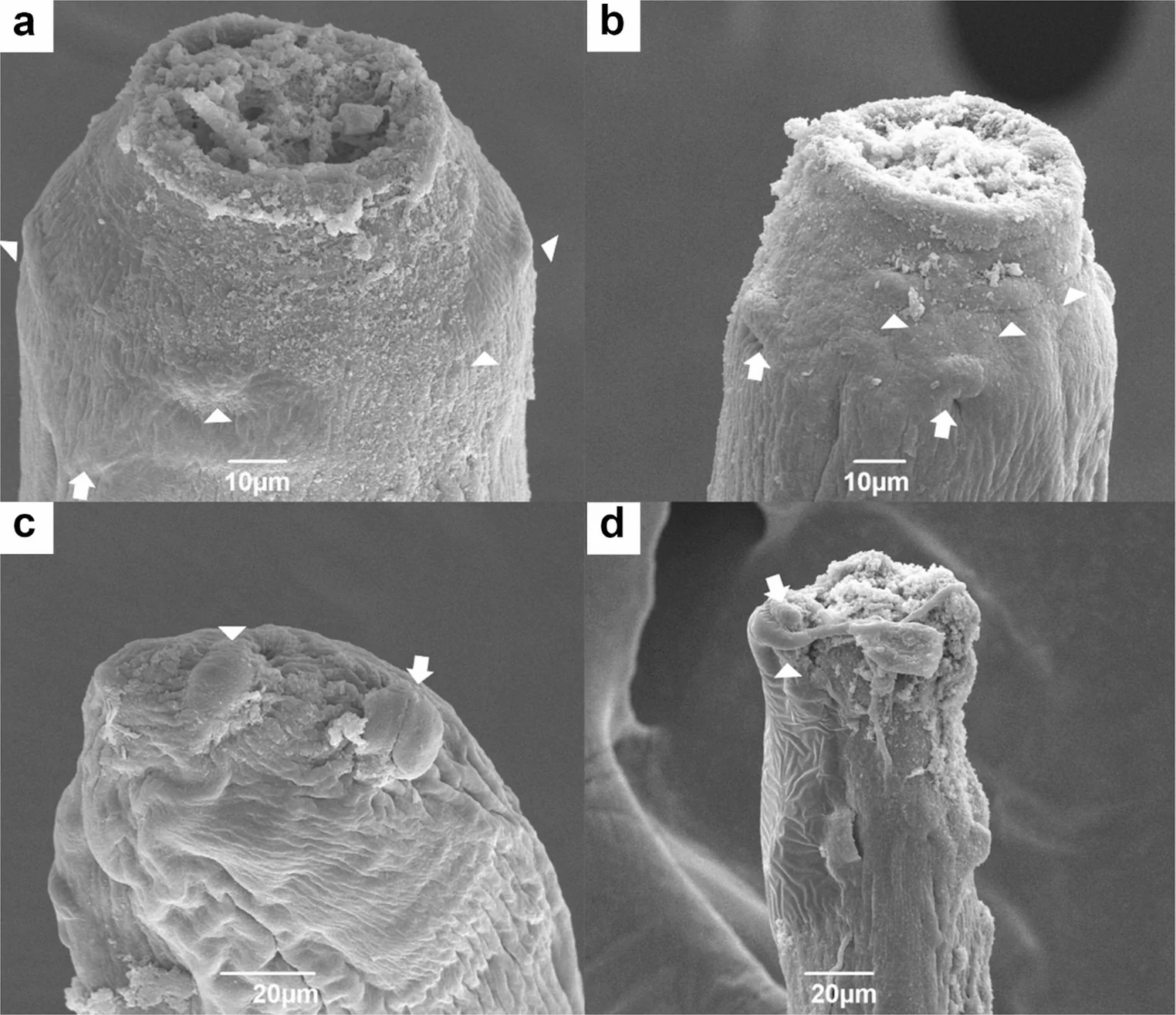
Scanning electron photomicrographs of* Stenurus globicephalae*. **(a)** Apical view of the cephalic end of a female. Note the circular oral opening, the inner circle of papillae (arrowheads), and the outer circle of four submedian papillae (arrows). **(b)** Sub-apical view of a male, showing a similar arrangement of cephalic papillae. **(c)** Caudal end of a female in ventral view, showing the prominent vulva (arrow) and anus (arrowhead). **(d)** Caudal end of a male in ventrolateral view, detailing the stout lateral ray (arrow) and medial papilla (arrowhead) of the copulatory bursa with mucus

The nematodes found in the tympanic bullae were identified as *Stenurus globicephalae* based on a set of key diagnostic taxonomic features observed under both light and scanning electron microscopy (Figs. 5 and 6) that are well-documented in the literature (Zylber et al. 2002; Saldaña et al. 2022). Specifically, the anterior end of both sexes presented a funnel-shaped buccal cavity and a circular oral opening surrounded by cephalic papillae arranged in an inner and an outer circle. The caudal end of the males was characterized by a copulatory bursa with stout lateral ray, medial papilla, short, curved, and equal spicules, and a simple gubernaculum. The caudal end of the females was distinguished by the prominent vulva, surrounded by a characteristic spherical cuticular swelling, an elongated vaginal sphincter, and the anus. This set of morphological features, together with the morphometric analysis (Table 1), confirmed the species identification.

**Table 1:** Comparative morphometric measurements of *Stenurus globicephalae* reported in different hosts and localities

Characteristic	Present Study		Zylber et al. (2002)		Saldaña et al. (2022)		Shamsi et al. (2024)	
Host	*Peponocephala electra*		*Pseudorca crassidens*		*Globicephala melas*		*Globicephala macrorhynchus*	
Site of Infection	Tympanic bullae		Cranial air sinuses		Pterygoid sinuses		Blowhole	
Reference	-		Zylber et al. (2002)		Saldaña et al. (2022)		Shamsi et al. (2024)	
-	**Male**	**Female**	**Male**	**Female**	**Male**	**Female**	**Male**	**Female**
MALE	(*N* = 2)	(*N* = 6)	(*N* = 60)	(*N* = 60)	(*N* = 23)	(*N* = 84)	(*N* = 6)	(*N* = 9)
Body length (mm)	29.9–31.1 (30.5)	37.7–45.3 (41.9)	28.1–31.7 (29.9)	38.9–47.9 (43.4)	25.2 ± 3.9	44.2 ± 2.6	25.1–35.0	35.0-49.7
Body width (µm)	365–398 (382)	351–423 (389)	320–420 (384)	560–660 (616)	-	-	435–490	625–748
Buccal capsule (µm)	31–33 (32) (ant.) / 90–98 (94) (post.)	38–44 (41) (ant.) / 81–99 (92) (post.)	25–40 (32) (ant.) / 55–75 (67) (post.)	40–65 (45) (ant.) / 75–90 (80) (post.)	-	-	45–60 (width)	60–81 (width)
Esophagus (µm)	588–613 (601) x 92–99 (96)	607–741 (677) x 113–138 (126)	500–600 (536) x 80–100 (91)	520–700 (610) x 95–130 (101)	-	-	503–666 (length)	558–762 (length)
Nerve ring (DFAE, µm)	275–291 (283)	181–219 (201)	160–200 (172)	170–250 (198)	-	-	190–245	218–354
Excretory pore (DFAE, µm)	594–625 (610)	459–545 (502)	475–550 (516)	490–650 (539)	-	-	358–476	490–585
Spicules (µm)	128–141 (135)	-	110–135 (116)	-	-	-	99–165	-
Gubernaculum (µm)	32–34 (33)	-	40–65 (50)	-	-	-	18–27	-
Vulva (dist. from anus, µm)	-	45–55 (49)	-	40–75 (56)	-	-	-	51 − 45

### Discussion and conclusion

In this study, we documented the pathological and parasitological findings of a case of severe nematodal otitis media in a MHW stranded in Galinhos, Northeastern Brazil, focusing on the anatomopathological findings of the tympanic apparatus, as well as the microscopic and ultrastructural morphology of the ear parasites, contextualizing these findings within the current literature.

Parasites with tropism for the cranial sinuses and ear structures are of particular importance in cetaceans due to their potential to cause significant dysfunction of acoustic perception and sound processing, thereby interfering with echolocation, which is a critical function for cetacean navigation, prey detection, social interactions and spatial orientation (Moss et al. 2023), and could result in aberrant behavior that leads to stranding (Mann et al. 2010). Otalgia (ear pain) is also likely present in such cases, as the ear possesses a complex and highly sensitive neural network involving branches of cranial nerves V (trigeminal), VII (facial), IX (glossopharyngeal), and X (vagus), as well as branches of the cervical plexus (C1, C2, and C3) (Kim 2023).

In Brazil, 36 records of MHWs were documented along the northern and northeastern coasts, spanning from Amapá to Bahia, between 1994 and 2023 (Freitas et al. 2025). Of these records, 77.8% (*n* = 28) were related to stranding events and 22.2% (*n* = 8) from at-sea sightings validated by photos. Similarly to our study, a fatal infection by *S. globicephalae* in *P. electra* was previously described in the country (Carvalho et al. 2010). In that case, the authors associated the high parasitic burden with neurological dysfunction characterized by disorientation, suggesting that the parasitic infection was linked to the stranding and death. In our study, behavioral data prior to death were not available, but apart from poor body condition, severe infection of the tympanic bulla by *S. globicephalae* was the main lesion identified during the post-mortem examination, since histopathology did not reveal other infectious lesions (e.g., viral or bacterial) that could explain the death of the animal. This leads us to hypothesize that clinical signs associated with this infection were present and may have contributed to the stranding and death of the animal.

The pathogenesis of otitis media and interna caused by parasites such as *Stenurus* spp., *Nasitrema* spp., and *Crassicauda* spp. involves traumatic injury resulting from mechanical compression and friction of the epithelium, which triggers persistent mucosal inflammation and epithelial adaptive changes (Phillips and Suepaul 2017; Febronio et al. 2021; Saldaña et al. 2022). As demonstrated by the case here reported, bone lesions are more common in infections by *S.globicephalae* (Zylber et al. 2002) and *Crassicauda* (Zucca et al. 2004), whereas *Nasitrema* infections are characterized by a more intense inflammatory response, typically granulomatous, surrounding flukes and thick-walled pigmented eggs. In addition, *Nasitrema* spp. have the capacity to migrate to the central nervous system, leading to fatal necrotizing meningoencephalitis in the most severe cases (Felipe-Jiménez et al. 2023; Suárez-González et al. 2024).

The lesions associated with nematodal otitis in cetaceans vary according to the parasite species involved and the specific site of infection, but they generally include epithelial hyperplasia, squamous metaplasia, ulceration, hemorrhage, and superficial necrosis, often accompanied by dense aggregates of worms (Wohlsein et al. 2019; Saldaña et al. 2022). Visualization of microscopic lesions in the soft tissues can be challenging, as the soft tissues of the middle ear are particularly fragile and difficult to adequately fix when specimens are simply immersed in formalin. We emphasize the importance of applying appropriate perfusion and tissue removal techniques, as described in the cetacean pathology literature (Morell and André 2009).

The life cycle of *Stenurus* is not fully elucidated (Pool et al. 2021; Saldaña et al. 2022). However, considering the general heteroxenous marine metastrongyloids cycle (Houde et al. 2003; Lehnert et al. 2010), it is believed that first-stage larvae (L1) are released in the feces of infected odontocetes and ingested by intermediate hosts, such as fish that feed on fecal material. In the intermediate host, the parasites develop into the third-stage larva (L3). Cetaceans, the definitive hosts, become infected when they ingest prey containing these larvae (Fischbach and Seguel 2023). Vertical transmission has also been suggested for marine metastrongyloids, including *S. globicephalae*, which has been reported in a neonate of *Grampus griseus*, indicating the possibility of transplacental or lactogenic infection (Pool et al. 2021).

Variations in morphometric measurements observed among *S. globicephalae* specimens from this study and those described in the literature, such as the length of Australian specimens and differences in gubernaculum size, may result from multiple factors, including host species, geographic origin, density dependency and specific infection sites, as observed in other pseudaliid nematode populations (Faulkner et al. 1998; Measures 2001; Pool et al. 2021; Saldaña et al. 2022). The physiological and immunological environments of different hosts may induce phenotypic plasticity or reflect local adaptations, while the specific microhabitat occupied by the parasite, whether in the cranial air sinuses, pterygoid sinuses, blowhole, or, as in the present case, the tympanic bullae, may influence morphological development due to spatial and nutritional constraints (Measures 2001; Poulin 2007; Saldanha et al. 2009). Biogeographic variation among parasite populations from the South Atlantic (Brazil and Uruguay) (Zylber et al. 2002), North Atlantic (Spain) (Saldaña et al. 2022), and Pacific Ocean (Australia) (Shamsi et al. 2024) may further contribute to these differences. The developmental stage of the parasite and the sample size analyzed may also influence morphometric variation, as methodological differences in fixation or preservation techniques may introduce artifacts that alter morphometric measurement (Knoff and Gomes 2012). However, the main morphological characteristics of the anterior region in both females and males, as well as the features of the male copulatory bursa and the posterior region of the female, are in accordance with other descriptions of *S. globicephalae* (Zylber et al. 2002; Saldaña et al. 2022; Shamsi et al. 2024).

Although several studies, including the present one, support the idea that heavy parasitic burdens in cetacean ear and cranial sinuses may have important clinical consequences, as previously discussed (Felipe-Jiménez et al. 2023; Saldaña et al. 2022; Suárez-González et al. 2024), the clinical significance of these infections are not totally elucidated and remains under discussion, because there were reports were animals harboring numerous parasites presented no associated gross lesions (Faulkner et al. 1998), or alterations in body condition, indicating preservation of essential physiological functions (Morell et al. 2017).

This case report underscores the relevance of auditory parasitic infection by *S. globicephalae* as an important differential diagnosis among potential causes of death in melon-headed whales stranded along the Brazilian coast and emphasizes the critical value of applying appropriate and systematic necropsy techniques to ensure accurate recognition and characterization of associated lesions. Our results reinforce the importance of evaluating the tympanic apparatus during cetacean necropsies. To the best of the authors’ knowledge, this study provides the most complete image-based morphological characterization of such infections in cases diagnosed in Brazil.

## Data Availability

No datasets were generated or analysed during the current study.

## References

[CR1] Abollo E, López A, Gestal C et al (1998) Macroparasites in cetaceans stranded on the northwestern Spanish Atlantic coast. Dis Aquat Organ 32:227–231. 10.3354/dao032227 9676247

[CR2] Braulik GT, Taylor BL, Minton G et al (2023) Red-list status and extinction risk of the world’s whales, dolphins, and porpoises. Conserv Biol 37:e14090. 10.1111/cobi.14090 37246556

[CR4] Carvalho VL, Bevilaqua CML, Iñiguez AM et al (2010) Metazoan parasites of cetaceans off the northeastern coast of Brazil. Vet Parasitol 173:116–122. 10.1016/j.vetpar.2010.06.023 20638793

[CR6] Esposito E, Oliviero M, Iaccarino D et al (2025) Post Mortem Findings of Cetaceans Stranded Along the Campania Coast from 2016 to 2022. Animals 15(12):1812. 10.3390/ani15121812 40564361 PMC12189110

[CR7] Faulkner J, Measures LN, Whoriskey FG (1998) *Stenurus minor* (Metastrongyloidea: Pseudaliidae) infections of the cranial sinuses of the harbour porpoise, *Phocoena phocoena*. Can J Zool 76:1209–1216. 10.1139/z98-057

[CR8] Febronio AM, Boos GS, Batista RL et al (2021) Crassicaudiasis in three geographically and chronologically distant Cuvier’s beaked whales (*Ziphius cavirostris*) stranded off Brazil. Int J Parasitol Parasites Wildl 16:262–269. 10.1016/j.ijppaw.2021.10.010 34824971 PMC8605309

[CR9] Felipe-Jiménez I, Fernández A, De Quirós YB et al (2023) Cerebral nasitremiasis in a Blainville’s beaked whale (*Mesoplodon densirostris*) stranded in the Canary Islands. Res Vet Sci 158:56–64. 10.1016/j.rvsc.2023.03.009 36934640

[CR11] Fischbach JR, Seguel M (2023) A systematic review of the diversity and virulence correlates of metastrongyle lungworms in marine mammals. Parasitology 150:1178–1191. 10.1017/S0031182023001014 37859401 PMC10801380

[CR10] Freitas DC, Bassoi M, Da Silva IVB (2025) Melon-Headed Whale (*Peponocephala electra*) Occurrence in the Equatorial Mid-Atlantic Ocean With Descriptions of Their Whistles. Mar Mammal Sci 42:e70094. 10.1111/mms.70094

[CR13] Houde M, Measures LN, Huot J (2003) Experimental transmission of *Pharurus pallasii* (Nematoda: Metastrongyloidea), a lungworm of the cranial sinuses of the beluga whale (*Delphinapterus leucas*), to fish. Can J Zool 81:364–370. 10.1139/z03-016

[CR14] IUCN (2025) Peponocephala electra. The IUCN Red List of Threatened Species 2025. International Union for Conservation of Nature, Gland. Available at: https://www.iucnredlist.org/species/18596/50358835. Accessed 19 May 2026

[CR15] Kim C-H (2023) Otalgia and Geniculate Neuralgia. In: Park K, Cho KR (eds) Trigeminal Neuralgia. Springer Nature Singapore, Singapore, pp 249–258. 10.1007/978-981-19-9171-4_26

[CR16] Knoff M, Gomes DC (2012) Metodologia básica para coleta e processamento de helmintos parasitos. In: Molinaro EM, Caputo LFG, Amendoeira MRR (eds) Conceitos e métodos para a formação de profissionais em laboratórios de saúde. EPSJV/Fiocruz, Rio de Janeiro, pp 251–282

[CR17] Kuwamura M, Sawamoto O, Yamate J et al (2007) Pulmonary vascular proliferation and lungworm (*Stenurus ovatus*) in a bottlenose dolphin (*Tursiops turncatus*). J Vet Med Sci 69:531–533. 10.1292/jvms.69.531 17551228

[CR19] Lehnert K, Raga J, Siebert U (2005) Macroparasites in stranded and bycaught harbour porpoises from German and Norwegian waters. Volume contents Dis Aquat Org 64:265–269. 10.3354/dao064265 15997825

[CR21] Lehnert K, von Samson-Himmelstjerna G, Schaudien D, Bleidorn C, Wohlsein P, Siebert U (2010) Transmission of lungworms of harbour porpoises and harbour seals: Molecular tools determine potential vertebrate intermediate hosts. Int J Parasitol 40:845–853. 10.1016/j.ijpara.2009.12.008 20123100

[CR20] Lehnert K, Randhawa H, Poulin R (2017) Metazoan parasites from odontocetes off New Zealand: new records. Parasitol Res 116:2861–2868. 10.1007/s00436-017-5573-0 28799088

[CR22] Lodi L, Siciliano S, Capistrano L (1990) Mass stranding of *Peponocephala electra* (Cetacea, Globicephalinae) on Piracanga Beach, Bahia, northeastern Brazil. Sci Rep Cetacean Res 1:79–84

[CR23] Mann D, Hill-Cook M, Manire C et al (2010) Hearing loss in stranded odontocete dolphins and whales. PLoS ONE 5:e13824. 10.1371/journal.pone.0013824 21072206 PMC2972210

[CR24] Measures LN (2001) Lungworms of marine mammals. In: Pybus MJ, Monismith SW, Kocan AA (eds) Parasitic diseases of wild mammals, 2rd edn. Iowa State University, Ames (IA), pp 230–257

[CR25] Miranda A, Luna F, Sousa G et al (2020) Guia de ilustrado de identificação de cetáceos e sirênios do Brasil–ICMBio/CMA. Instituto Chico Mendes de Conservação da Biodiversidade & Centro Nacional de Pesquisa e Conservação de Mamíferos Aquáticos, Brazil

[CR26] Morell M, André M (2009) Cetacean ear extraction and fixation protocol. University of British Columbia. https://www.zoology.ubc.ca/files/Ear_extraction_and_fixation_protocol_UBC.pdf. Accessed 22 March 2026

[CR27] Morell M, Lehnert K et al (2017) Parasites in the inner ear of harbour porpoise: cases from the North and Baltic Seas. Dis Aquat Organ 127:57–63. 10.3354/dao03178 29256428

[CR28] Moss CF, Ortiz ST, Wahlberg M (2023) Adaptive echolocation behavior of bats and toothed whales in dynamic soundscapes. J Exp Biol 226:jeb245450. 10.1242/jeb.245450 37161774 PMC10184770

[CR30] Perryman WL, Danil K (2018) Melon-headed whale: *Peponocephala electra*. In: Würsig B, Thewissen JGM, Kovacs KM (eds) Encyclopedia of marine mammals. Elsevier, pp 593–595

[CR31] Phillips ACN, Suepaul R (2017) *Nasitrema* Species: A Frequent Culprit in Melon-Headed Whale (*Peponocephala electra*) Strandings in Trinidad. Aquat Mamm 43(5):547–557

[CR32] Pinto RM, Knoff M, Gomes DC, Noronha D (2011) Nematodes from mammals in Brazil: an updating. Neotrop Helminthol 5:139–183

[CR33] Pool R, Chandradeva N, Gkafas G et al (2020) Transmission and predictors of burden of lungworms of the striped dolphin (*Stenella coeruleoalba*) in the Western Mediterranean. J Wildl Dis 56:186–191 31295079

[CR34] Pool R, Romero-Rubira C, Raga JA et al (2021) Determinants of lungworm specificity in five cetacean species in the western Mediterranean. Parasit Vectors. 10.1186/s13071-021-04629-1 33845871 PMC8042974

[CR35] Poulin R (2007) Evolutionary Ecology of Parasites. Princeton University Press

[CR36] Reidenberg JS, Laitman JT (2025) Review of respiratory anatomy adaptations in whales. Anat Rec (Hoboken) 308:1179–1213. 10.1002/ar.25597 39487584

[CR38] Roca-Monge K, González-Barrientos R, Suárez-Esquivel M et al (2022) Age and Sexual Maturity Estimation of Stranded Striped Dolphins, *Stenella coeruleoalba*, Infected with *Brucella ceti*. Oceans 3:494–508. 10.3390/oceans3040033

[CR39] Saldaña A, López C, López A et al (2022) Specificity of *Stenurus* (Metastrongyloidea: Pseudaliidae) infections in odontocetes stranded along the north-west Spanish coast. Int J Parasitol Parasites Wildl 19:148–154. 10.1016/j.ijppaw.2022.09.002 36133958 PMC9483633

[CR40] Saldanha I, Leung TLF, Poulin R (2009) Causes of intraspecific variation in body size among trematode metacercariae. J Helminthol 83:289–293. 10.1017/S0022149X09224175 19216824

[CR41] Seibel H, Beineke A, Siebert U (2010) Mycotic otitis media in a harbour porpoise (*Phocoena phocoena*). J Comp Pathol 143:294–296. 10.1016/j.jcpa.2010.03.002 20643415

[CR42] Shamsi S, Moravec F, Barton DP (2024) Occurrence of *Stenurus globicephalae* (Nematoda: Pseudaliidae) in the blowhole of *Globicephala macrorhynchus* (Cetacea: Delphinidae) in Tasmania, Australia. Dis Aquat Organ 158:115–122. 10.3354/dao03791 38717060

[CR43] Suárez-González Z, González JF, Arbelo M et al (2024) Parasitic infections in stranded whales and dolphins in canary islands (2018–2022): an update. Animals 14:3377. 10.3390/ani14233377 39682343 PMC11640159

[CR44] Temple AJ, Langner U, Berumen ML (2024) Management and research efforts are failing dolphins, porpoises, and other toothed whales. Sci Rep 14:7077. 10.1038/s41598-024-57811-7 38528092 PMC10963780

[CR45] Tomo I, Kemper CM, Lavery TJ (2010) Eighteen-year study of South Australian dolphins shows variation in lung nematodes by season, year, age class, and location. J Wildl Dis 46:488–498. 10.7589/0090-3558-46.2.488 20688641

[CR47] Wang Z-T, Supin AY, Akamatsu T et al (2021) Auditory evoked potential in stranded melon-headed whales (*Peponocephala electra*): With severe hearing loss and possibly caused by anthropogenic noise pollution. Ecotoxicol Environ Saf 228:113047. 10.1016/j.ecoenv.2021.113047 34861441

[CR48] Wohlsein P, Seibel H, Beineke A et al (2019) Morphological and pathological findings in the middle and inner ears of harbour porpoises (*Phocoena phocoena)*. J Comp Pathol 172:93–106. 10.1016/j.jcpa.2019.09.005 31690422

[CR49] Wunschimann A, Frese K, Müiller G et al (2001) Evidence of infectious diseases in harbour porpoises (*Phocoena phocoena*) hunted in the waters of Greenland and by-caught in the German North Sea and Baltic Sea. Vet Rec 148:715–720. 10.1136/vr.148.23.715 11430682

[CR50] Zucca P, Di Guardo G et al (2004) Use of computer tomography for imaging of *Crassicauda grampicola* in a Risso’s dolphin (*Grampus griseus*). J Zoo Wildl Med 35(3):391–394. 10.1638/02-098 15526896

[CR51] Zylber MI, Failla G, Le Bas A (2002) *Stenurus globicephalae* Baylis et Daubney, 1925 (Nematoda: Pseudaliidae) from a false killer whale, *Pseudorca crassidens* (Cetacea: Delphinidae), stranded on the coast of Uruguay. Mem Inst Oswaldo Cruz 97:221–225. 10.1590/S0074-02762002000200015 12016448

